# Artificial SEI for Superhigh‐Performance K‐Graphite Anode

**DOI:** 10.1002/advs.202003639

**Published:** 2021-02-08

**Authors:** Qian Liu, Apparao M. Rao, Xu Han, Bingan Lu

**Affiliations:** ^1^ State Key Laboratory of Advanced Design and Manufacturing for Vehicle Body College of Mechanical and Vehicle Engineering Hunan University Changsha 410082 China; ^2^ Department of Physics and Astronomy Clemson Nanomaterials Institute Clemson University Clemson SC 29634 USA; ^3^ School of Physics and Electronics Hunan Provincial Key Laboratory of Multi‐electron based Energy Storage Devices Hunan University Changsha 410082 China

**Keywords:** artificial inorganic SEI films, commercial graphite, initial Coulombic efficiency, potassium ion batteries

## Abstract

Although graphite with its merits of low cost, abundance, and environmental friendliness is a potential anode material for potassium ion batteries (PIBs), it suffers from a limited cycle life due to a severe decomposition of the solid electrolyte interface (SEI) in organic electrolytes. Herein, a simple and viable method is demonstrated for the first time through which an ultra‐thin, uniform, dense, and stable artificial inorganic SEI film can be prepared on commercial graphite anodes and used with traditional carbonate electrolytes to achieve PIBs with long‐cycle stability and high initial Coulombic efficiency (ICE). Specifically, such commercial graphite anodes exhibit a long‐term cycling stability for more than 1000 cycles at 100 mA g^−1^ (a reversible capacity of around 260 mAh g^−1^) and a high average CE (around 99.9%) in traditional carbonate electrolytes with no discernable decay in capacity. More importantly, the commercial graphite anodes with the artificial inorganic SEI film in traditional carbonate electrolytes can deliver a high ICE of 93% (the highest ICE ever reported for PIBs anodes until now), which improves the performance of the PIB full cell. Considering the high ICE and long cycle stability performance, this study can promote the rapid deployment of PIBs on a commercial scale.

## Graphical abstract

An ultra‐thin, uniform, dense, and stable artificial inorganic solid electrolyte interface (SEI) film is prepared on commercial graphite anodes and used in traditional carbonate electrolytes to achieve potassium ion batteries (PIBs) with long‐cycle stability and high initial Coulombic efficiency. The proposed artificial SEI method can promote the rapid deployment of PIBs on a commercial scale.

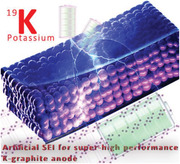

The lithium ion battery (LIB) is the workhorse of present day energy storage devices, and is used in several devices ranging from the mobile phones to electric cars. While LIBs are reliable, scalable and exhibit high energy and power densities, they suffer from the dwindling lithium resources.^[^ ^1^, ^2^, ^3^, ^4^, ^5^ ^]^ As such, alternate battery chemistries, such as the potassium ion (PIB) or the sodium ion (NIB) batteries have come into vogue as they can exhibit similar electrochemical performance as the LIBs; and relative to Li, both Na and K are abundantly present in the Earth's crust.^[^ ^6^, ^7^, ^8^, ^9^, ^10^ ^]^ The standard electrode potentials (vs SHE) of Na^+^ and K^+^ are −2.71 and −2.93 V, respectively which are close to the standard electrode potential of Li^+^ of −3.04 V, suggesting that the NIBs and PIBs could exhibit a comparable discharge voltage and energy density as the LIBs.^[^ ^9^, ^10^, ^11^, ^12^, ^13^, ^14^, ^15^, ^16^, ^17^, ^18^, ^19^, ^20^, ^21^, ^22^, ^23^, ^24^, ^25^, ^26^, ^27^, ^28^, ^29^ ^]^

Due to its low cost and good chemical stability, graphite is the preferred choice for anodes in a commercial LIB, which has also been explored widely for use as an anode in NIBs and PIBs^.[^ ^30^, ^31^, ^32^ ^]^ While it remains challenging to intercalate sodium ions into graphite anodes, several reports have shown that potassium ions readily intercalate into graphite to form potassium‐graphite intercalation compounds and deliver a high reversible capacity of 273 mAh g^−1^.^[^ ^12^, ^33^ ^]^ Nevertheless, the graphite anodes of PIBs in organic electrolytes suffer from severe electrolyte decomposition, which leads to a low initial Coulombic efficiency (ICE) and an inferior cycle life.^[^ ^34^ ^]^ Typically, the safety and cycle stability of a battery are determined by its anode's characteristics, and its passivation layer plays a critical role in the battery performance.^[^ ^35^ ^]^ However, the graphite anode with an uneven solid‐electrolyte interphase (SEI) film (for example, in traditional carbonate‐based electrolytes for PIBs) is mainly composed of organic elements, which are known to react strongly with water and air, resulting in a poor long cycle stability and potential safety risks.^[^ ^36^ ^]^ Considering the low ICE and its importance in commercial rechargeable batteries, it is crucial to develop graphite anodes with appropriate protective layer for use in PIBs with traditional carbonate‐based electrolytes that have been embraced by the battery industry.

Herein, a novel method is presented for preparing ultra‐thin, uniform, dense and stable artificial inorganic SEI films on commercial graphite anodes for PIBs. Notably, it is shown that such inorganic SEI films can be controllably designed on the surface of a graphite anode when it is kept in contact with a K metal foil and soaked in a high concentration inorganic potassium bis(fluorosulfonyl)imide (KFSI) in 1,2‐dimethoxyethane (DME) electrolyte (abbreviated as KFSI‐DME electrolyte). The artificial SEI layer forms spontaneously due to the favorable energetics of the materials used in the method. The high concentration KFSI‐DME electrolyte acts as the source as well as the transporting medium for the potassium ions, which diffuse into the graphite anode. As delineated below, the artificial SEI film realized by this method is inorganic in nature, which also mitigates the otherwise large consumption of potassium ions by the organic SEI film in a full‐cell battery. As a result, the commercial graphite anodes with the artificial SEI could deliver a reversible capacity of 260 mAh g^−1^ for more than 1000 cycles with a capacity retention as high as 100% at 100 mA g^−1^ – this is the longest cycle stability exhibited by any PIB graphite anode in the traditional carbonate‐based electrolytes until now. More remarkably, the graphite anode with the artificial inorganic SEI exhibits a high ICE of 93% – this is the highest ICE reported yet for any PIB anodes. Importantly, the full battery with the artificial inorganic SEI could also exhibit excellent performance, confirming that the graphite anode with such artificial inorganic SEI represents a promising technological advancement for commercial applications.

The artificial inorganic SEI film prepared via the simple one‐step method (**Figure** **1**a) forms spontaneously on the graphite anode (Figure 1b). Typically, the graphite anode is kept in contact with the K metal foil and soaked in highly concentrated KFSI‐DME electrolyte for a few hours. The relatively higher standard potential of the K metal compared to that of graphite drives the decomposition of KFSI and the subsequent diffusion of K ions into graphite. It is well known that the K ions diffuse into graphite with a standard electrode potential of −2.93 eV. The K ions first adsorb on the surface of the graphite anode, and then intercalate into the graphite anode to form the intercalation compound KC_8_ (the calculated value is 0.51 eV for KC_8_).^[^ ^33^ ^]^ For comparison, artificial organic SEI film was also prepared by bringing a graphite anode into contact with a K metal foil and soaking them together in 0.8 m potassium hexafluorophosphate (KPF_6_) in the ethyl carbonate (EC): ethyl methyl carbonate (EMC) (1:1, *v:v*) electrolyte, or 0.8 m KPF_6_‐ EC:EMC.

**Figure 1:**
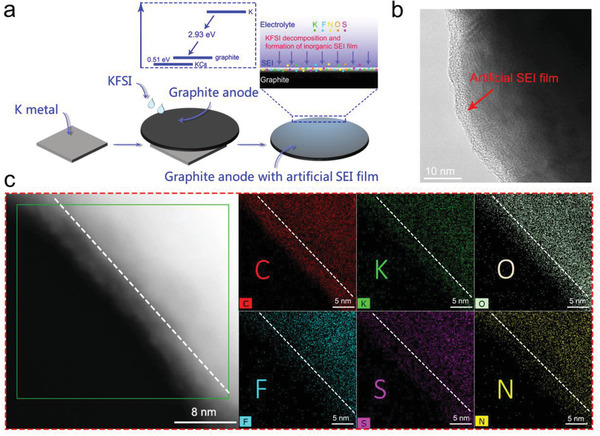
Schematic, HRTEM image and the element maps of the artificial SEI film. a) Process that spontaneously forms an artificial SEI on a graphite anode when it is placed in contact with a K metal foil and soaked in a KFSI‐DME electrolyte; b) The HRTEM image of an artificial SEI film formed on a graphite anode; c, The element maps of the artificial SEI film on graphite. The corresponding XPS spectra are discussed in Figure S6 in the Supporting Information.

To decipher the role of the electrolyte concentration in the formation of the artificial SEI, graphite anodes were kept in contact with the K metal foil in varying concentrations (1, 2, 3, and 4 m) of the KFSI‐DME electrolyte for the same duration of time of 5 h (Figure S1, Supporting Information). The ICE gradually increased with increasing concentration of the electrolyte until it saturated in 3 m KFSI‐DME eletrolyte, implying that the anode was potassiated to near saturation during its contact with the K metal foil in > 3 m electrolytes. Their corresponding discharge capacities are shown in Figure S2 in the Supporting Information. In another experiment, the graphite anodes were kept in contact with the K metal foil and soaked in 3 m KFSI‐DME electrolyte for 5, 10, 15, and 20 h, respectively. As evident in Figure S3 in the Supporting Information, their ICE did not always increase with longer soak time which implied a threshold time of ≈15 h for the spontaneous formation of the artificial SEI in the 3 m KFSI‐DME electrolyte. The corresponding discharge capacities are presented in Figure S4 in the Supporting Information. In Figure 1b and Figure S5 (Supporting Information), it is evident that an ultra‐thin, uniform, dense SEI film (≈3 nm) was formed on the surface of the graphite anode. Elemental mapping of this artificial SEI film before cycling revealed the presence of distributed C, O, N, F, S, and K elements in the artificial SEI film (Figure 1c). To futher investigate the composition of the artificial SEI films, XPS analysis was performed before cycling. The XPS full surveys of the artificial SEI films exhibited the obvious peaks of C 1s, K 2p, K 2s, O 1s, F 1s, S 2s, S 2p, and N 1s (Figure S6, Supporting Information), and the high‐resolution F1s, S 2p, and N1s XPS (Figure S6, Supporting Information) spectra confirmed the decomposition of KFSI. Consistent with other reports in the literature, our XPS analysis and element mapping thus indicated that the artificial SEI film is mainly composed of inorganic elements.^[^ ^17^, ^34^, ^36^ ^]^

Commercial graphite with artificial inorganic (3 m KFSI‐DME), artificial organic (0.8 m KPF_6_‐EC:EMC), traditional (0.8 m KPF_6_‐EC:EMC without the soaking step involved) and traditional with the addition of 10 wt% FEC in 0.8 m KPF_6_‐EC:EMC SEI films were prepared (corresponding schematics are shown in Figure S7 in the Supporting Information) and their performance as PIB anodes was evaluated. The charge/discharge profiles for the 2nd cycle to the 100th cycle of the graphite anode with artificial SEI proved much superior relative to the traditional SEI film (Figure S8, Supporting Information). Clearly, as evident in **Figure** **2**a: i) the graphite anode with an artificial inorganic SEI exhibits a better cycle stability than the one with the traditional SEI in the organic electrolyte; and ii) the battery with artificial organic SEI film (0.8 m KPF_6_‐EC:EMC) suffers a rapid capacity fade (Figure S9, Supporting Information). Additionally, coin cells using graphite as anodes and 0.8 m KPF_6_‐EC:EMC with 10 wt% FEC as the electrolyte, were also assembled and the cycle performance of these half cells was investigated (Figure 2a; Figure S10, Supporting Information). As seen clearly in Figure S10 in the Supporting Information, the addition of FEC does not improve the battery performance, and at 100 mA g^−1^ it exhibits a rapid capacity decay. Specifically, using graphite anode with the artificial inorganic SEI film, a reversible capacity of 260 mAh g^−1^ over 1000 cycles with a CE of around 100% at 100 mA g^−1^ was recorded. Notably, it exhibits a high ICE of 93%, while the graphite anode with the traditional SEI film exhibits a low ICE of 58%. A scatter plot comparing the ICE of the graphite anode with traditional and artificial SEI films is shown in Figure S11 in the Supporting Information. The excellent performance in the latter is due to the presence of the uniform, dense and stable artificial inorganic SEI film. It is well known that the KFSI is more thermodynamic reactive, and likely to decompose easily and form SEI film, which protects the graphite anode surface and prevents further decomposition of the organic electrolyte. The charge/discharge profiles for the 100th to 1000th cycle of the graphite anode with an artificial SEI film are shown in Figure 2b, while the capacity for the 900th to 1000th cycle are shown in Figure 2c. As evidient in Figure 2a, the capacity retention is about 100%. Lastly, the charge/discharge profiles from 400th to 1000th cycles of graphite anode with artificial SEI film are shown in Figures S12–S14 in the Supporting Information.

**Figure 2:**
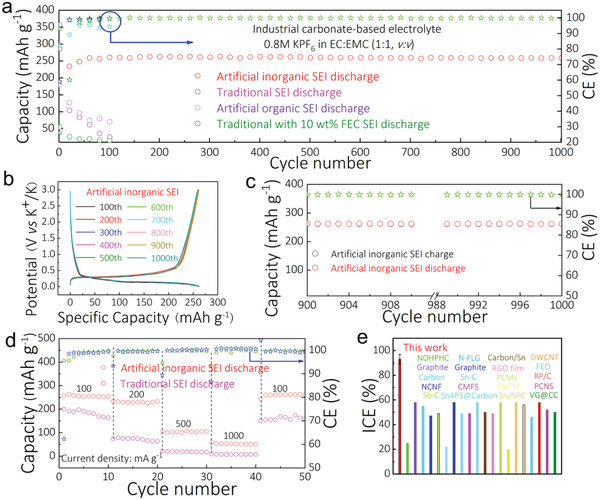
Electrochemical performance of graphite with an artificial SEI film. a) The cycle performance of the graphite anode at 100 mA g^−1^ with artificial inorganic SEI film (3 m KFSI‐DME), traditional SEI film, artificial organic SEI film (0.8 m KPF_6_‐EC:EMC), and traditional SEI film prepared with the addition of 10 wt% FEC in 0.8 m KPF_6_‐EC: EMC; b) The charge‐discharge profiles from 100th to 1000th cycle of the graphite anode with artificial SEI film; c) The cycle performance from 900th to 1000th cycle of the graphite anode with artificial SEI film at 100 mA g^−1^. The charge and discharge data points overlap, implying a CE% of 100%; d, The rate performance of the graphite anode with the artificial SEI (CE represented by green stars) and traditional SEI (CE represented by blue stars) films; e, Comparison of the ICE of the graphite anode with artificial SEI film with other reported anodes.(^[^ ^7^, ^9^, ^10^, ^12^, ^31^, ^34^, ^37^, ^38^, ^39^, ^40^, ^41^, ^42^, ^43^, ^44^ ^]^)

The rate performance of graphite anode with the artificial and traditional SEI films is compared (100–1000 mA g^−1^) in Figure 2d. The battery with artificial SEI film could deliver a discharge capacity of 260 mAh g^−1^ at a current density of 100 mA g^−1^, and could still deliver discharge capacities of 220, 110, and 60 mAh g^−1^ at 200, 500, and 1000 mA g^−1^, respectively. It is noteworthy that the capacity recovered to 260 mAh g^−1^ when the current density was restored to 100 mA g^−1^, confirming an excellent rate performance. By contrast, the graphite anode with the traditional SEI film delivers relatively low capacities of 170 and 20 mAh g^−1^ at 100 and 500 mA g^−1^, respectively. Remarkably, the graphite anode with the artificial SEI film also exhibits a high ICE of 93% as evident in Figure 2a, which is the highest ICE among all reported anodes for PIBs (Figure 2e).^[^ ^7^, ^9^, ^10^, ^12^, ^31^, ^34^, ^37^, ^38^, ^39^, ^40^, ^41^, ^42^, ^43^, ^44^ ^]^ Collectively, it can be inferred that the artificial inorganic SEI film prevents the organic electrolyte (DME, EC, EMC) from further decomposition, which improves the electrochemical performance of the anode, thereby setting a high benchmark for ICE of PIBs (Figure 2e), or any other battery at large.

XPS analysis was performed to investigate the compositions of the artificial and traditional SEI films (**Figure** **3**) after the 5th and the 50th cycle. The XPS full surveys of the artificial and traditional SEI films exhibit five obvious peaks of C 1s, K 2p, K 2s, O 1s, and F 1s (Figure 3a).^[^ ^34^ ^]^ Furthermore, three new peaks assigned to S 2s, S 2p, and N 1s for the graphite anode with the artificial SEI film are also evident, which arise from the decomposition of the inorganic KFSI. The artificial and traditional SEI films exhibit different peaks in the high‐resolution C 1s, O 1s, and F 1s XPS data (Figure 3b–d).^[^ ^36^ ^]^ In Figure 3b, the artificial and traditional SEI films both exhibit peaks corresponding to C—C/C=C, C—O, C=O, and O—C=O. Nevertheless, the relative amounts of C—O, C=O, and O—C=O for the traditional SEI film are higher than that for the artificial SEI film. This may be on account of the decomposition of the organic electrolyte (EC, EMC), implying that the graphite anode in KPF_6_‐based traditional organic electrolyte suffers from a severe solvent decomposition. Moreover, the high resolution O 1s spectra (Figure 3c) exhibits peaks corresponding to C=O, C—O, and O—C=O for the graphite anode electrode with the traditional SEI film. The presence of the RO‐K^[^ ^34^ ^]^ peak also confirmed the serious decomposition of KPF_6_‐based traditional organic electrolyte. The high resolution F 1s XPS spectra revealed the presence of KF on the surface of the graphite electrode with artificial SEI film, while lesser KF was found on graphite electrode surface with the traditional SEI film, further demonstrating that the artificial SEI film is inorganic in nature (Figure 3d). This conclusion is consistent with the presence of other inorganic components on the surface of the graphite anode (Figure 3e,f). Collectively, the XPS analysis found that the main ingredients of artificial SEI film are inorganic in nature, while for the traditional SEI film, they are mainly organic in nature.

**Figure 3:**
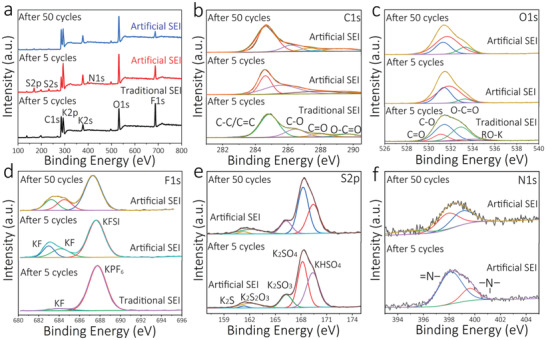
XPS analysis of graphite anodes with the artificial SEI film and traditional SEI film. a–d) the artificial SEI film and traditional SEI films after the 5th and 50th cycle. a) Full survey XPS; b) C 1s XPS; c) O 1s XPS; d) F 1s XPS; e,f) the artificial SEI films after the 5th and 50th cycle; e) S 2p XPS; f) N 1s XPS.

**Figure** **4**a,b illustrates the structural changes in the artificial and traditional SEI films after many cycles in the traditional carbonate electrolyte. As expected, the traditional SEI film on graphite anode exhibited a discontinuous, non‐dense and unstable film after many cycles. Due to the non uniform nature of the traditional organic SEI, some regions of the anode surface are devoid of the traditional organic SEI. As a result, the SEI film thickens locally on account of repeated SEI film formation, which then results in a rapid consumption of the organic electrolyte leading to a limited cycle life, and low ICE. In contrast, the structure of the artificial SEI film on graphite anode is uniform and extremely robust even after many cycles, which is primarily due to the presence of the inorganic SEI film on the graphite anode. We know that the KFSI is thermodynamically more reactive, and likely to decompose easily and form a highly stable SEI film, leading to high stability^.[^ ^36^ ^]^

**Figure 4:**
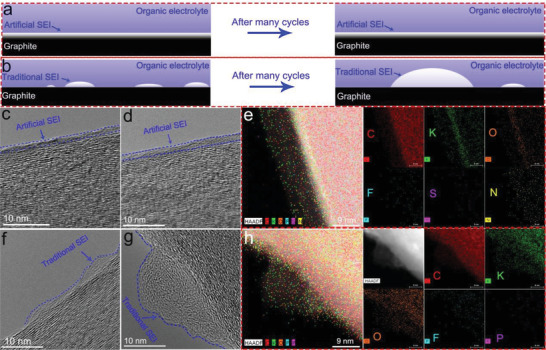
Schematic, HRTEM image and the element mappings of the artificial SEI film and traditional SEI film at different cycles. a,b) Schematic illustration of the artificial and traditional SEI films after many cycles; c,d) The HRTEM image of artificial SEI film on graphite anode. c) after 5 cycles; d) after 50 cycles; e) The elemental maps of the artificial SEI film on graphite after 50 cycles; f,g) The HRTEM image of traditional SEI film on graphite anode. f) after 5 cycles; g) after 50 cycles; h) The element maps of traditional SEI film on graphite after 50 cycles.

Transmission electron microscopy (TEM) further revealed the structure of the traditional and artificial SEI films (Figure 4; Figures S15 and S16, Supporting Information). In Figure 4c, an ultra‐thin, uniform, dense of SEI layers is evident, and even after 50 cycles the SEI layer is continuous and stable (Figure 4d). To further understand the elemental composition of the artificial SEI film on graphite anode, elemental maps were collected (Figure 4e) which reveal the presence of the C, O, N, F, S, and K inorganic elements that are distributed in the artificial SEI film. On the contrary, the traditional SEI film is discontinuous, non‐dense and unstable (Figure 4f) because the SEI film gets thicker after 50 cycles as evident in Figure 4g, which might be responsible for its inferior stability and limited cycle life. Also, the organic SEI film that was formed on the surface of the graphite anode (by soaking it and the K metal foil in 0.8 m KPF_6_‐EC:EMC) showed a discontinuous, non‐dense and unstable SEI film (Figure S16a, Supporting Information), and the SEI film becomes thicker after 50 cycles as shown in Figure S16b in the Supporting Information. Its elemental content of C, O, K, P, and F are presented in Figure 4h and Figure S17 (Supporting Information), indicating that the SEI film is mostly organic in nature. The corresponding energy spectra are shown in Figures S18 and S19 in the Supporting Information.

To investigate the influence of thickness variation of the artificial and traditional SEI films during their formation on the graphite anode, the electrochemical impedance spectroscopy (EIS) data of PIBs was collected after different cycles (0th, 5th, 10th, 30th, 50th) to monitor the changes in the interfacial impedance (Figures S20 and S21, Supporting Information). The diameter of the semicircle in Figure S20 in the Supporting Information increases constantly, revealing that the traditional SEI film on graphite is being formed continuously. On the other hand, the diameter of the semicircle in Figure S21 in the Supporting Information shows a gradual increase in the first five cycles, and then no obvious change in its diameter even after 50 cycles, revealing that the artificial SEI film is uniform, dense, and stable.

Lastly, we used a graphite anode with the artificial SEI film as anode and perylene‐3,4,9,10‐tetracarboxylic dianhydride (PTCDA)^[^ ^18^ ^]^ as the cathode and assembled a full cell to investigate the commercial potential of graphite anodes with artificial SEI films (**Figure** **5**; Figure S22, Supporting Information). During charge process, the K^+^ in the electrolyte moved toward the graphite anode and inserts into the graphite anode, the K^+^ inserts into the PTCDA cathode during discharge process. The cycle performance of PTCDA cathode is exhibited in Figure S23 in the Supporting Information. The typical charge/discharge profiles of the PTCDA or graphite half and full cells are shown in Figure 5a. The rate performance of the full cell (the specific capacities are calculated based on the active materials in both anode and cathode) is shown in Figure 5b – it delivered reversible discharge capacities of 82, 79, 76, 74, and 71 mAh g^−1^ at current densities of 20, 30, 50, 60, and 70 mA g^−1^, respectively. A capacity of 77 mAh g^−1^ was measured when the current density was restored to 20 mA g^−1^, demonstrating its superior rate performance. The full cell could easily power a LED (Figure 5b), and at a current density of 50 mA g^−1^, it exhibits high CE and discharge capacity of 61 mAh g^−1^ after 50 cycles (Figure 5c). In contrast, the full cell with traditional SEI film exhibits low CE and a rapid capacity decay, demonstrating that the artificial inorganic SEI film also improves the full battery performance, particularly in CE and long cycle stability performance. Collectivley, the full cell data bodes well for commercializing PIBs with anodes that contain the inorganic SEI films.

**Figure 5:**
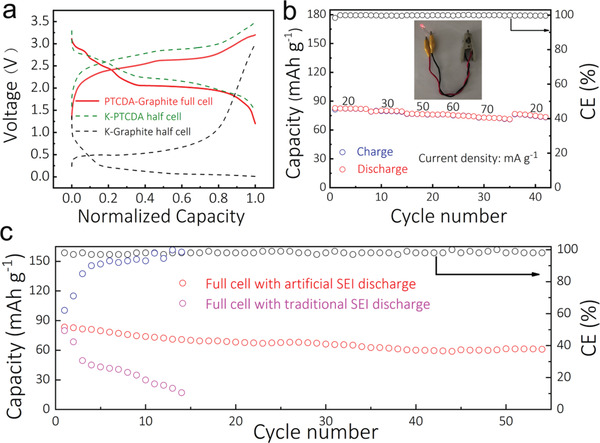
Full cell based on graphite anode and PTCDA cathode. a) The typical charge/discharge profiles of the half and full cells; b) Rate performance of the full cell with artificial SEI film, and an example of a LED powered by the full cell; c) Cycle stability of full cell with artificial SEI and traditional SEI at a current density of 50 mA g^−1^.

In summary, by using a one‐step method we successfully prepared an ultra‐thin, uniform, dense and stable artificial inorganic SEI film on graphite anodes for PIBs in traditional carbonate electrolytes. The PIBs exhibited excellent performance with a long cycle performance over 1000 cycles, a capacity retention as high as 100%, and a high ICE of 93% at 100 mA g^−1^. The artificial inorganic SEI film prevents the electrolyte from further decomposition during potassiation and depotassiation. This work provides a novel one‐step method for designing artificial inorganic SEI films for PIBs, especially for obtaining long‐cycle stability and high ICE using traditional carbonate electrolytes.

## Experimental Section

##### The Design of Artificial SEI Film—Artificial Inorganic SEI Film

The artificial inorganic SEI was prepared directly on graphite anode through a spontaneous reaction of K metal (Sigma‐Aldrich) with a high concentration of inorganic KFSI (Sigma‐Aldrich) in DME (BASF). The artificial inorganic SEI film formed spontaneously on the graphite anode surface when the graphite anode was kept in contact with a K metal foil and soaked in a high concentration inorganic KFSI in DME electrolyte. Graphite anodes were kept in contact with the K metal foil in varying concentrations (1, 2, 3, and 4 m) of the KFSI‐DME electrolyte for the same duration of time of 5, 10, 15, and 20 h, respectively. All experiments were conducted inside the glove box full of Ar gas with the content of water and oxygen lower than 0.5 ppm.

##### Artificial Organic SEI Film

The organic SEI was prepared on the graphite anode surface by soaking the graphite anode along with the K metal foil in organic electrolyte such as KPF_6_ (Sigma‐Aldrich) in EC:EMC (1:1, *v:v*). All experiments were conducted inside the glove box full of Ar gas with the content of water and oxygen lower than 0.5 ppm.

##### Synthesis of Heated PTCDA

The cathode material^[^ ^18^ ^]^ was prepared by heating PTCDA at 450 °C for 4 h with a heating rate of 5 °C min^−1^ under Ar.

##### Material Characterization

TEM (Tecnai F20) was used to study microstructure morphology of graphite anode, and ESCALAB 250Xi for the XPS measurements.

##### Electrochemical Measurements

Active materials (graphite anode and PTCDA cathode) were mixed with conductive carbon black, and carboxyl methyl cellulose with a weight ratio of 8:1:1. Next, the slurry was coated onto copper and aluminum foils with an areal density of 1.5 and 2.5 mg cm^−2^, respectively, the precision of the scale (Sartorius) is 0.01 mg, and then dried at 80 °C for over 10 h. Half cells (2032 type coin cells) were prepared using a potassium foil as the counter electrode, graphite as the anode electrode, and Whatman glass fibers (Whatman, thickness ≈ 1 mm) as the separator. Three types of electrolytes were used in this study: KFSI in DME (for the artificial inorganic SEI), KPF_6_ in EC:EMC (for the artificial organic SEI), and KPF_6_ in EC:EMC with 10 wt% FEC (to study the benefit of including FEC during the formation of traditonal SEI). The full cells were prepared using graphite anode with artificial SEI film, and PTCDA as the cathode, and 0.8 m KPF_6_ in EC: EMC as the electrolyte. After cycling the assembled full cell for 3 cycles in the voltage range 1.2–3.5 V, the data presented in Figure 5 was collected using a Neware BTS‐53 system. All cells were assembled in a glove box full of Ar gas with the content of water and oxygen lower than 0.5 ppm.

## Conflict of Interest

The authors declare no conflict of interest.

## Supporting information

Supporting Information

## Data Availability

Research data are not shared.
