# Supplementary material for: Artificial SEI for Superhigh‐Performance K‐Graphite Anode
Source: Adv Sci (Weinh). 2021 Feb 8;8(9):2003639. doi: 10.1002/advs.202003639 (PMC8097355; doi:10.1002/advs.202003639)
Supplement: Supplementary file 1 — Supporting Information [file ADVS-8-2003639-s001.pdf]

© 2021 Wiley-VCH GmbH

ADVANCED SCIENCE
Open Access

Supporting Information

for *Adv. Sci.,* DOI: 10.1002/advs.202003639

Artificial SEI for Super-high Performance K-Graphite Anode

*Qian Liu, Apparao M. Rao, Xu Han * and Bingan Lu **

Supporting Information

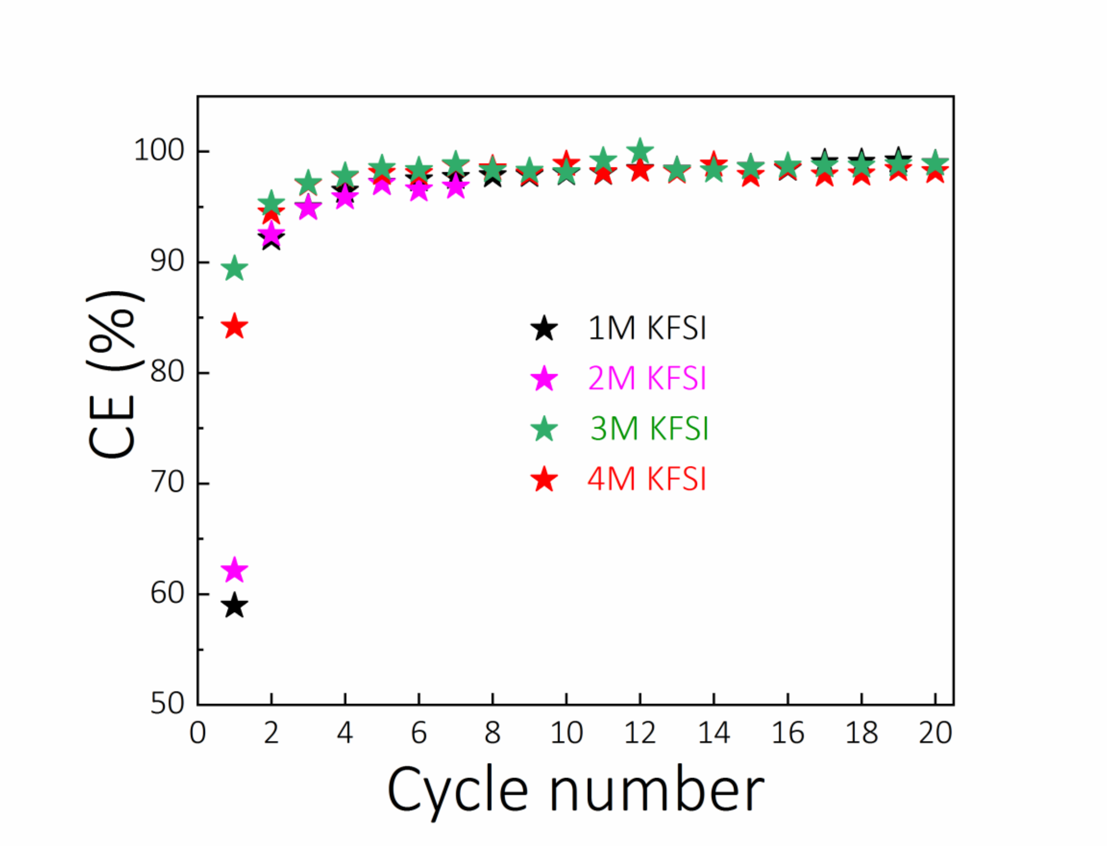

Figure S1. Comparison of the CE of graphite anodes whose artificial SEI films were prepared by soaking the anode and K metal foil in different concentrations (1M, 2M, 3M and 4M) of KFSI-DME electrolyte for 5 hours.

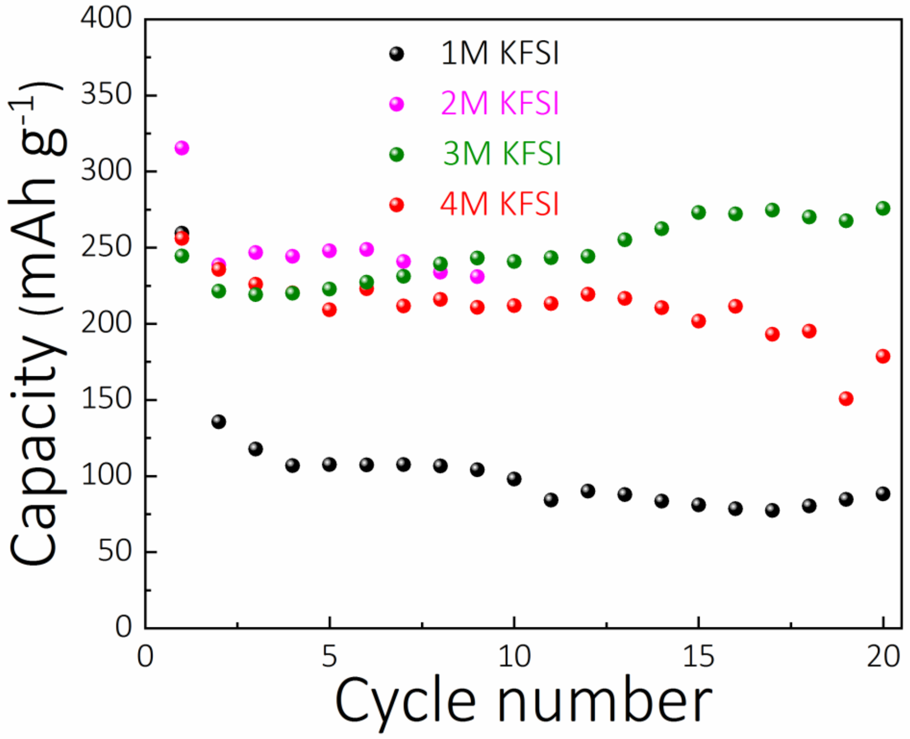

Figure S2. Comparison of the discharge capacities of the graphite anodes discussed in Figure S1.

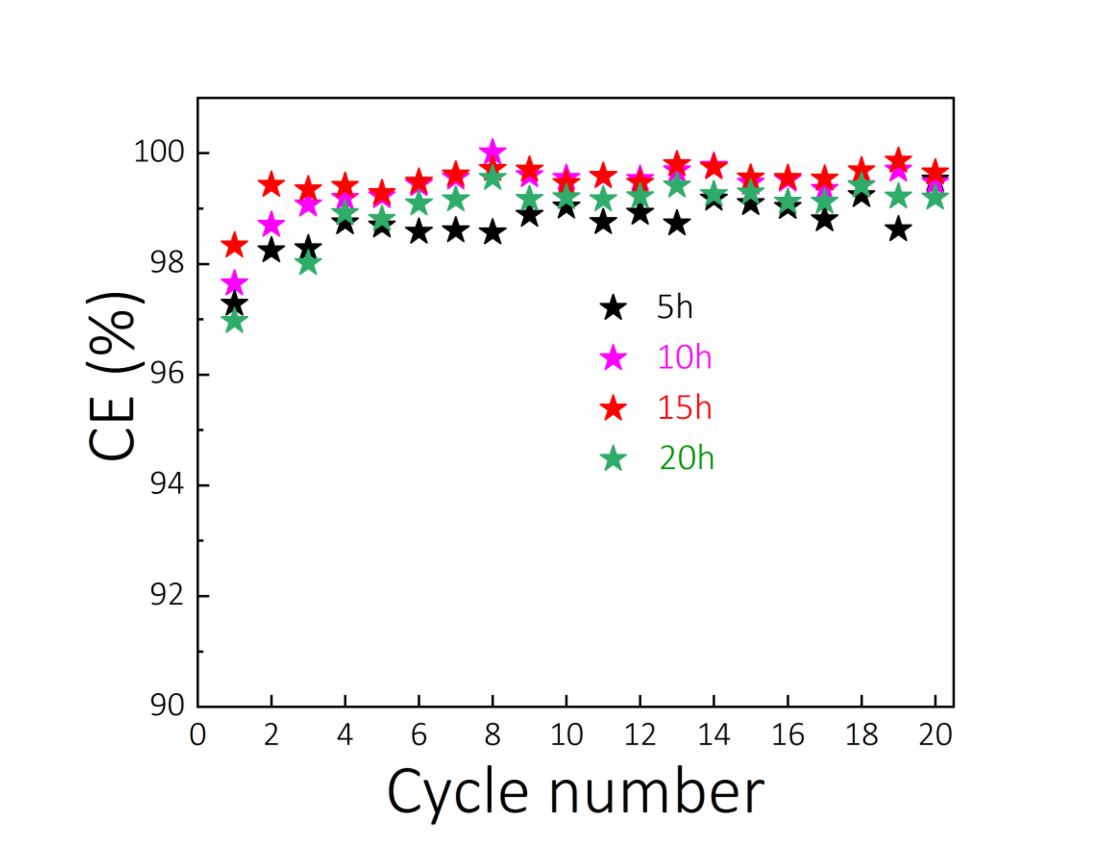

Figure S3. Comparison of the CE of graphite anodes that were kept in contact with the K metal foil in 3M KFSI-DME electrolyte for 5h, 10h, 15h and 20h.

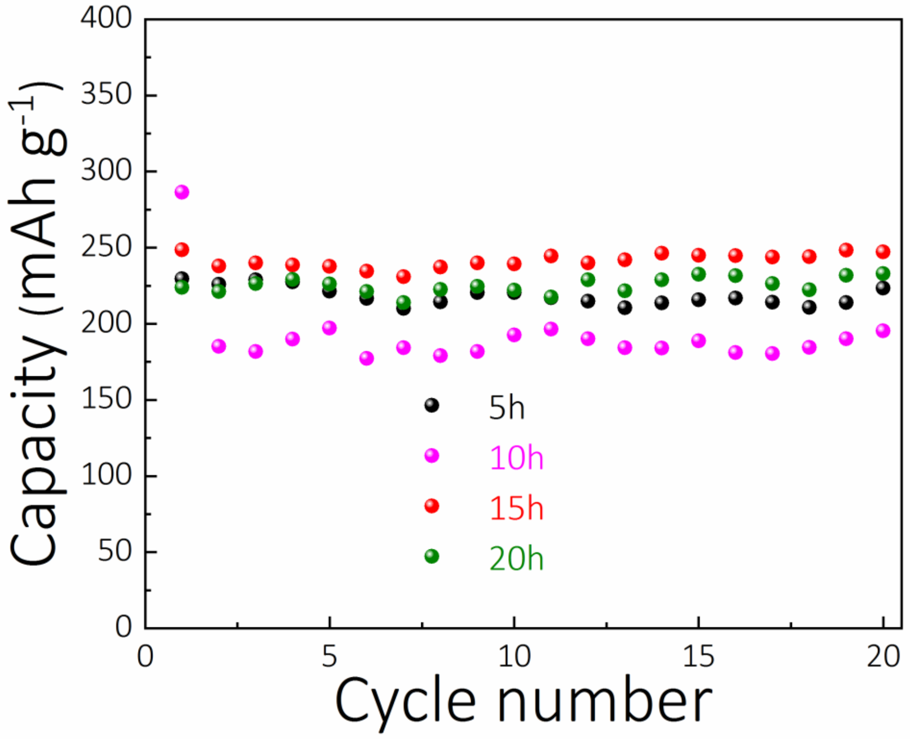

Figure S4. Comparison of the discharge capacities of the graphite anodes discussed in Figure S3.

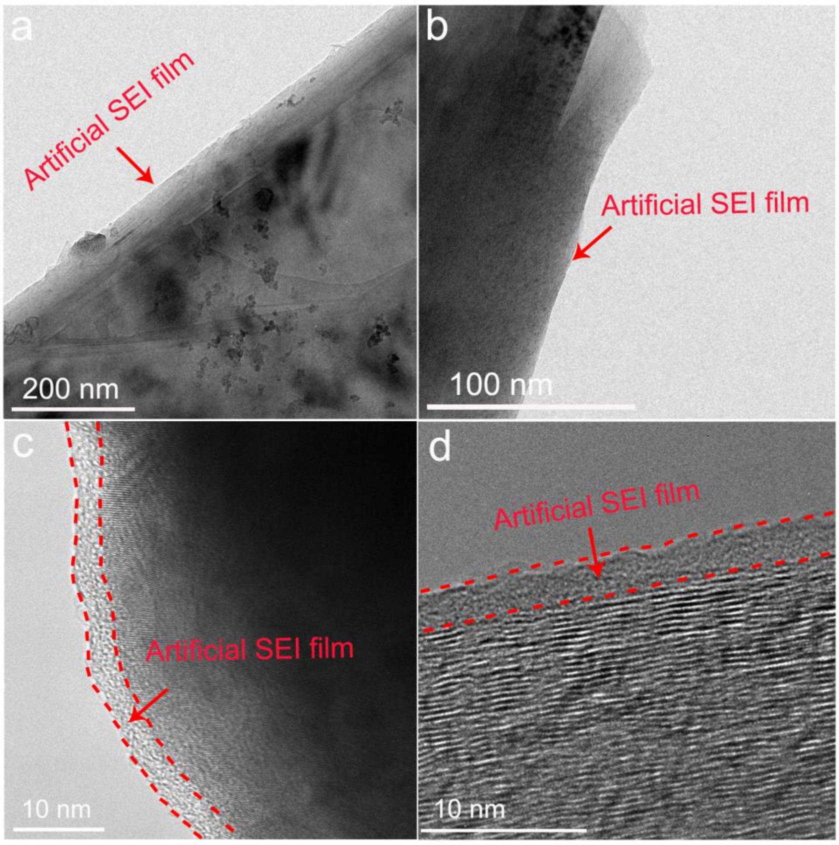

Figure S5. The TEM and HRTEM image of the artificial SEI film on the surface of a graphite anode.

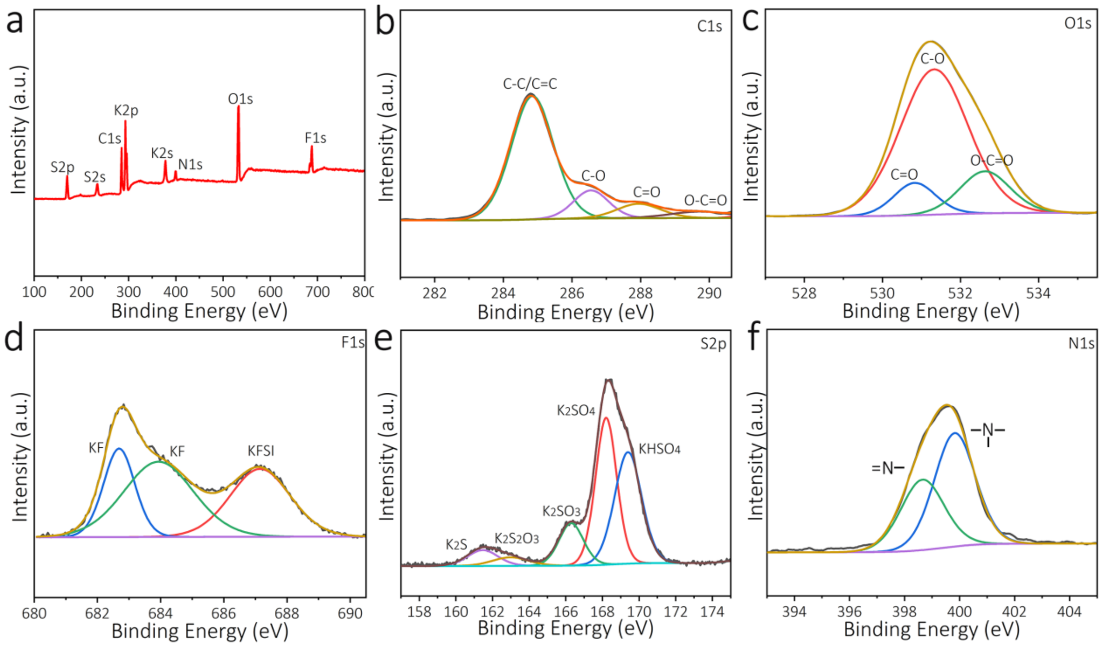

Figure S6. XPS analysis of graphite anode with the artificial SEI film before cycling. a, Full survey XPS; b, C 1s XPS; c, O 1s XPS; d, F 1s XPS; e, S 2p XPS; f, N 1s XPS.

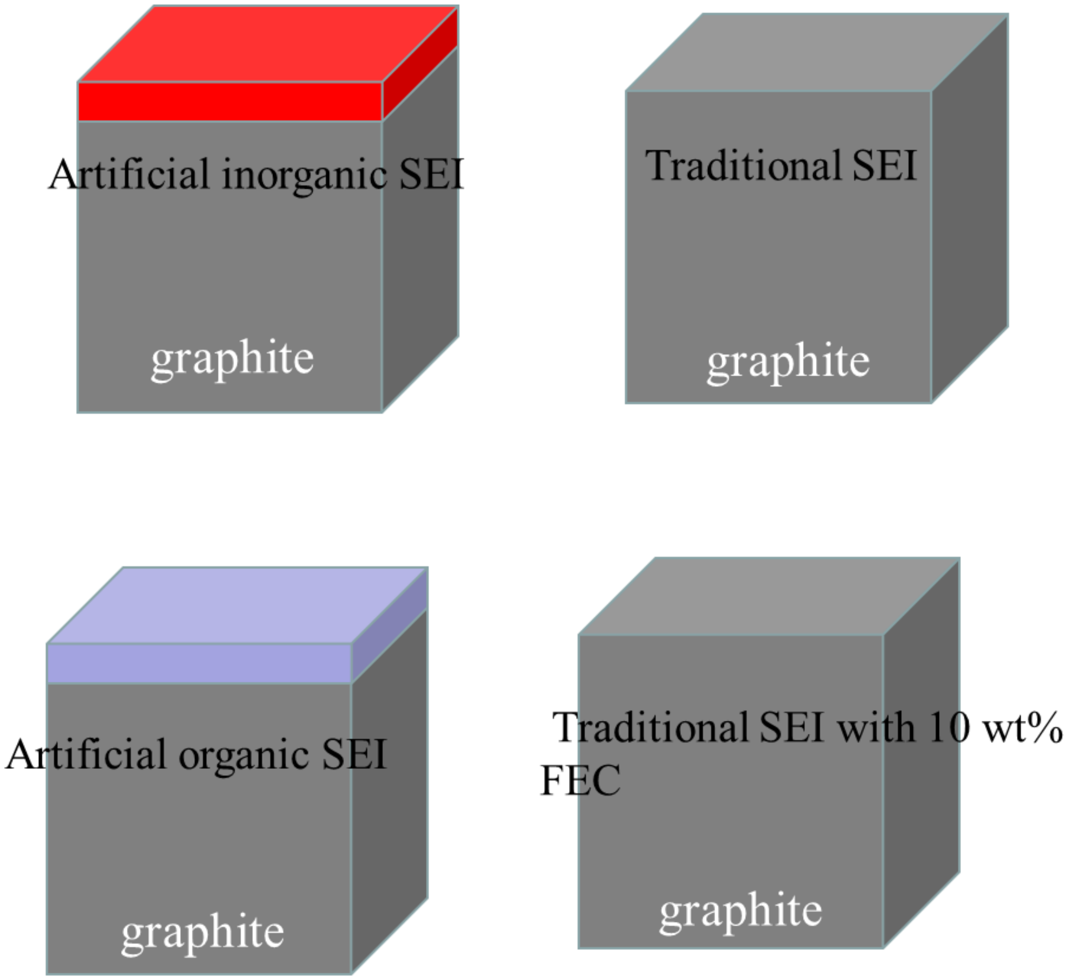

Figure S7. Corresponding schematics of graphite with artificial (3M KFSI-DME), artificial organic (0.8M KPF_6-EC:EMC), traditional (0.8M KPF_6-EC:EMC without the soaking step involved) and traditional with the addition of 10 wt% FEC in 0.8M KPF_6-EC: EMC SEI films.

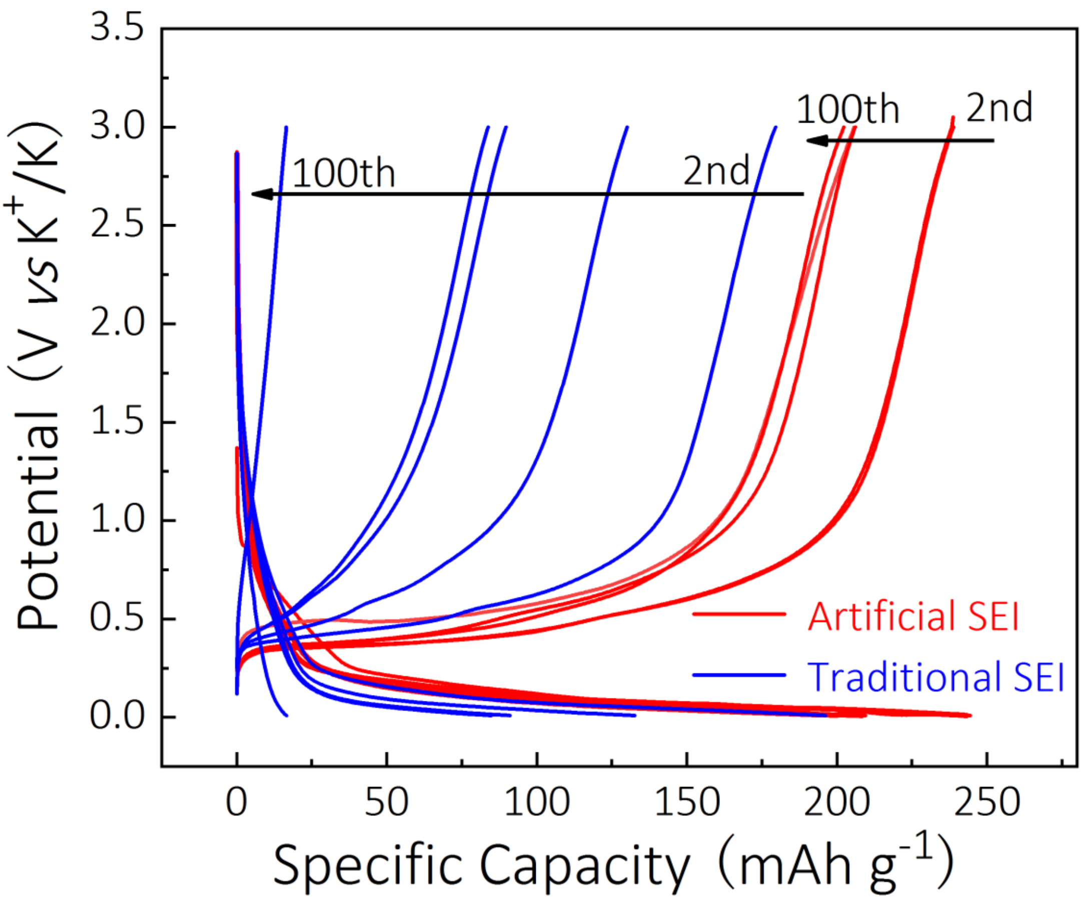

Figure S8. Comparison of the charge-discharge profiles of graphite anodes with artificial and traditional SEI films in 0.8M KPF_6-EC:EMC (1:1, *v*:*v*).

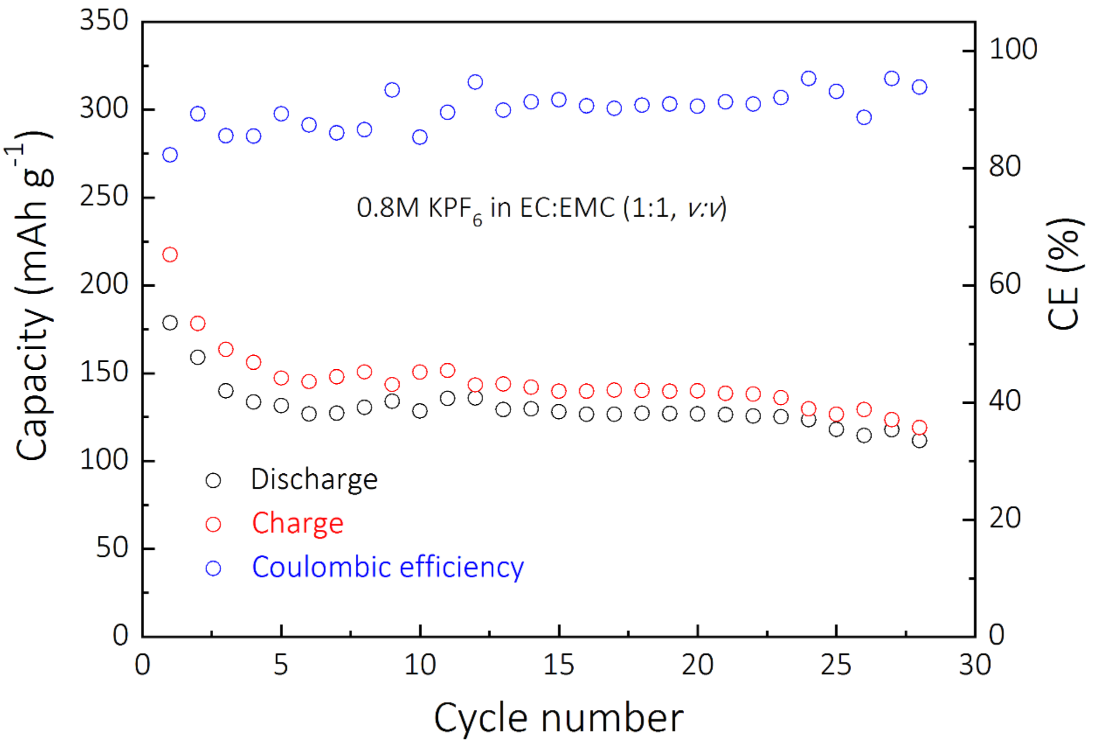

Figure S9.The cycle performance of the graphite anode with the artificial organic SEI film (KPF_6) at 100 mA g^{-1}.

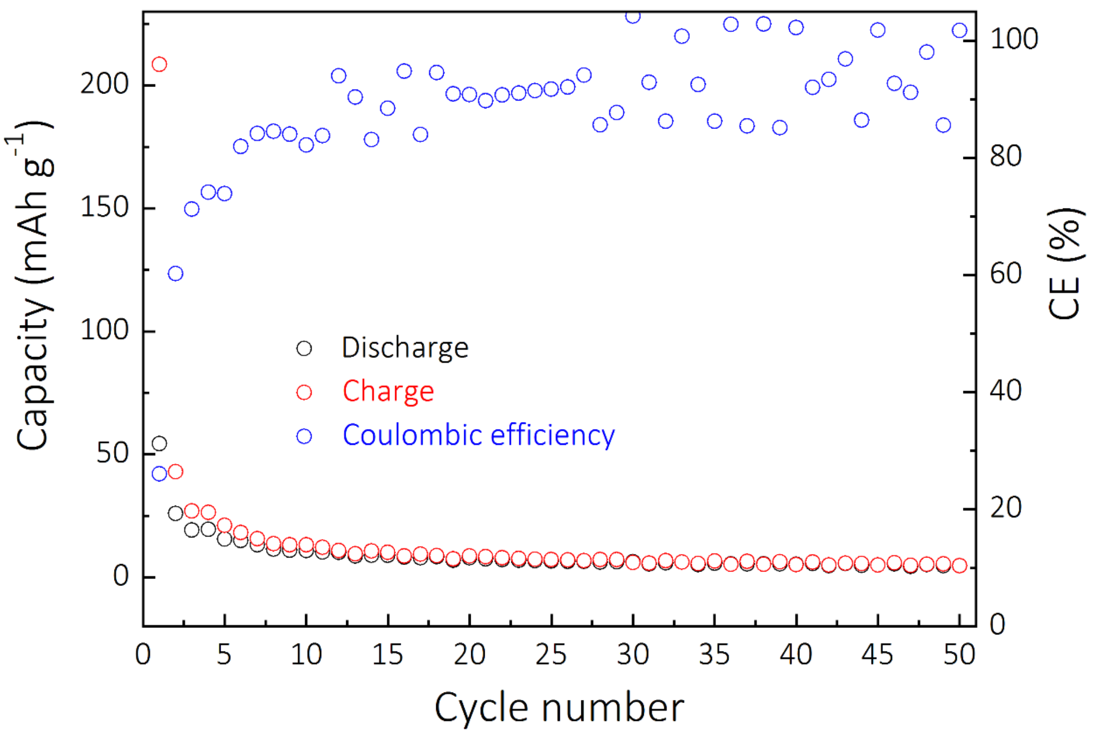

Figure S10. The cycle performance of the graphite anode with 0.8M KPF_6 in EC: EMC with 10 wt% FEC as electrolyte at 100 mA g^{-1}.

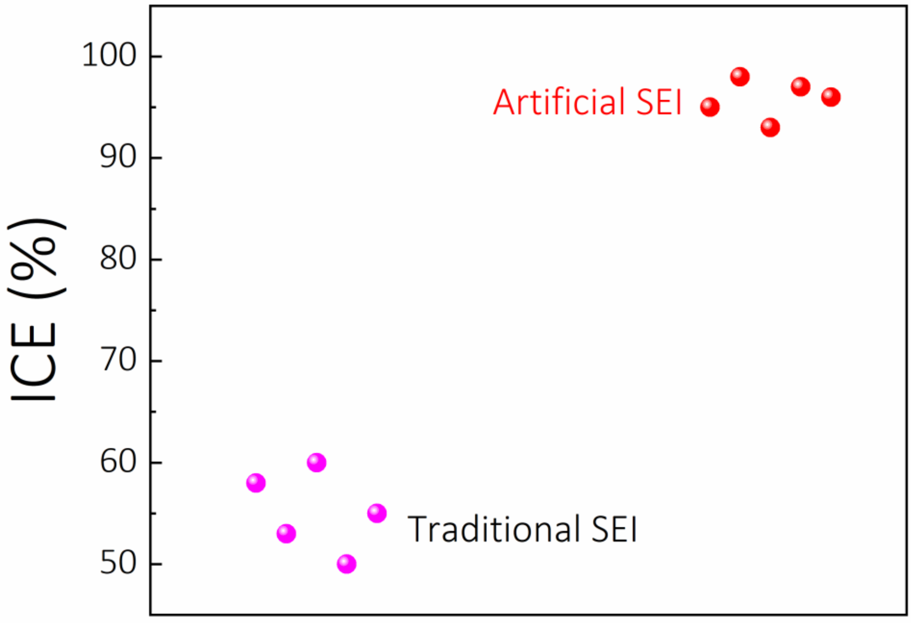

Figure S11. Scatter plot of the ICE of graphite anodes with traditional and artificial SEI films.

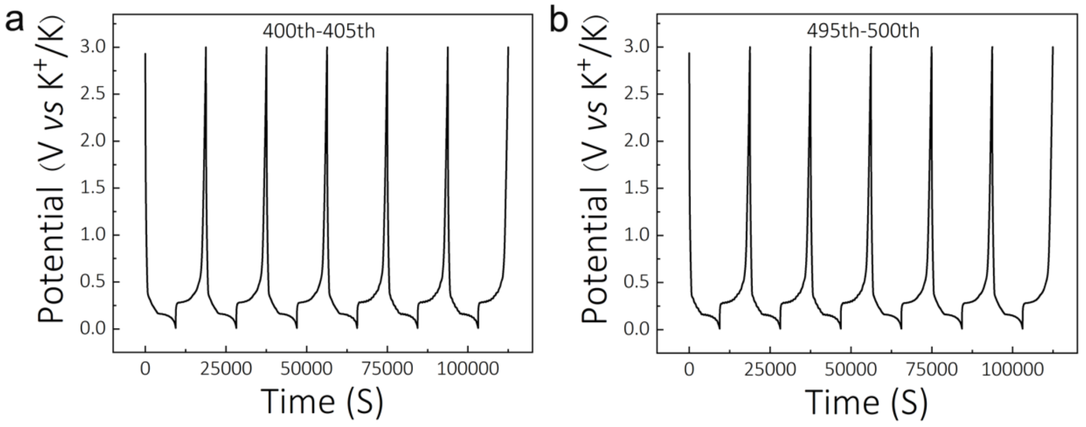

Figure S12. The charge-discharge profiles of the 400th to 500th cycle of the graphite anode with the artificial SEI film. Data for the (a) 400th to 405th cycle, and (b) 495th to 500th cycle.

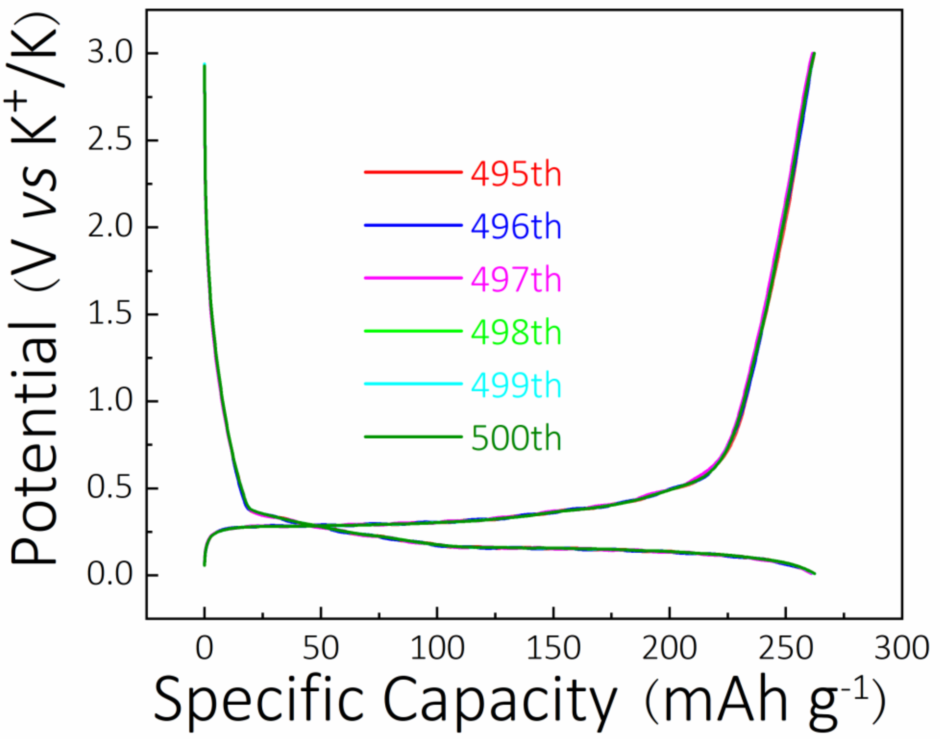

Figure S13. The charge-discharge profiles for the 495th to 500th cycle of the graphite anode with artificial SEI film.

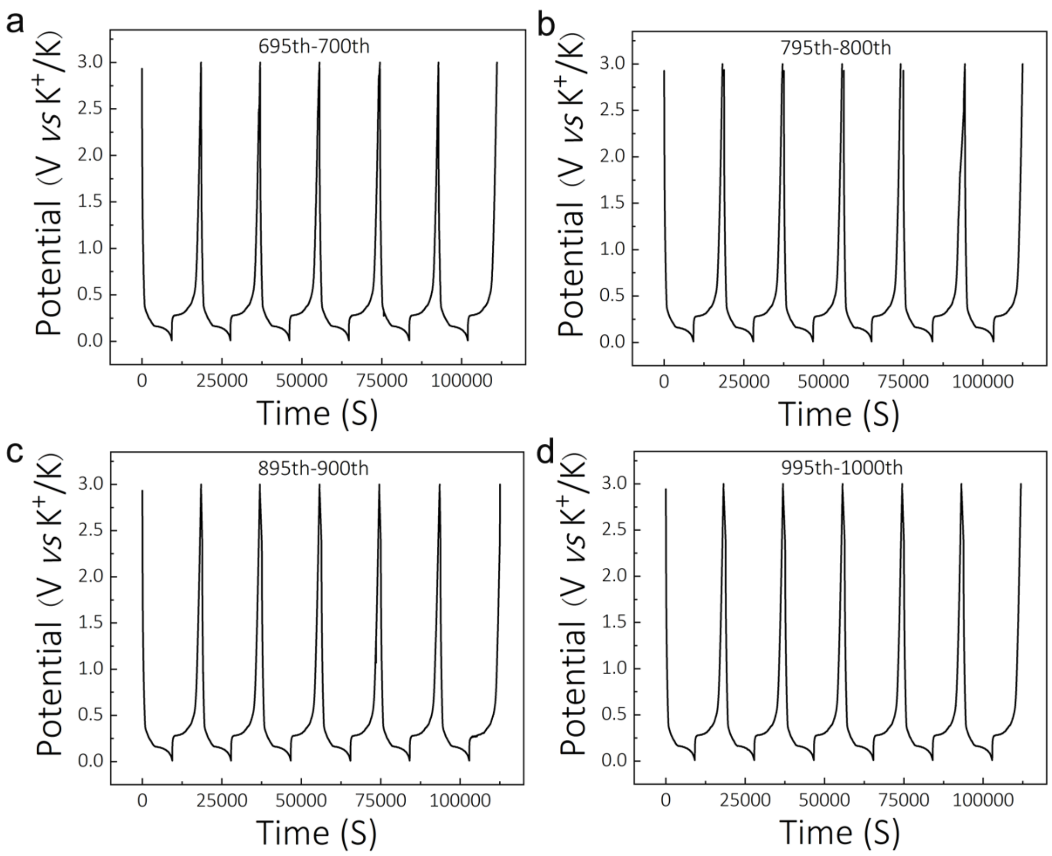

Figure S14. The charge-discharge profiles for the 695th to 1000th cycle of the graphite anode with the artificial SEI film. (a) 695th to 700th cycle, (b) 795th to 800th cycle, (c) 895th to 900th cycle, and (d) 995th to 1000th cycle.

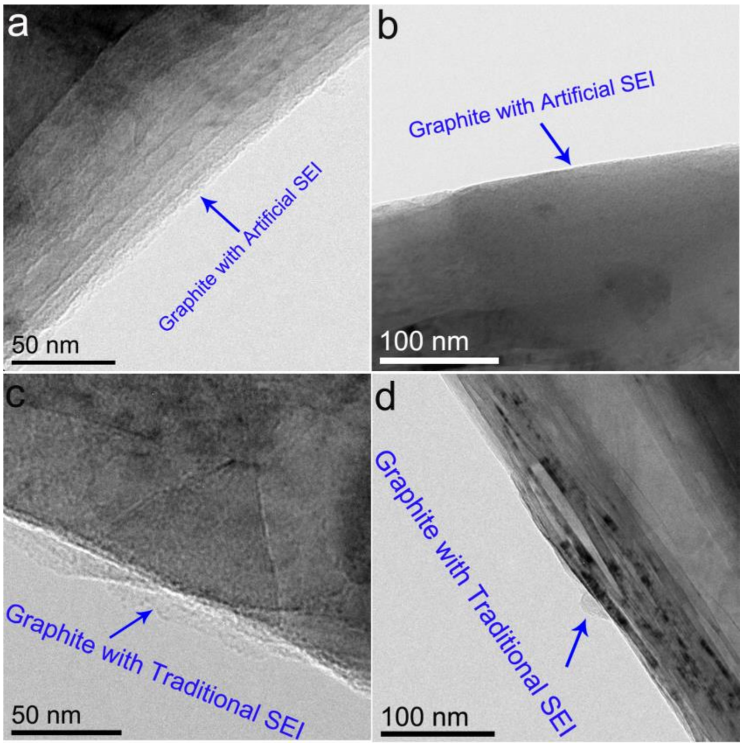

Figure S15. TEM image of the artificial and traditional SEI films at different cycles. (a-b) The TEM image of artificial SEI film on graphite anode. (a) after 5 cycles. (b) after 50 cycles. (c-d) The TEM image of traditional SEI film on graphite anode. (c) After 5 cycles. (d) After 50 cycles.

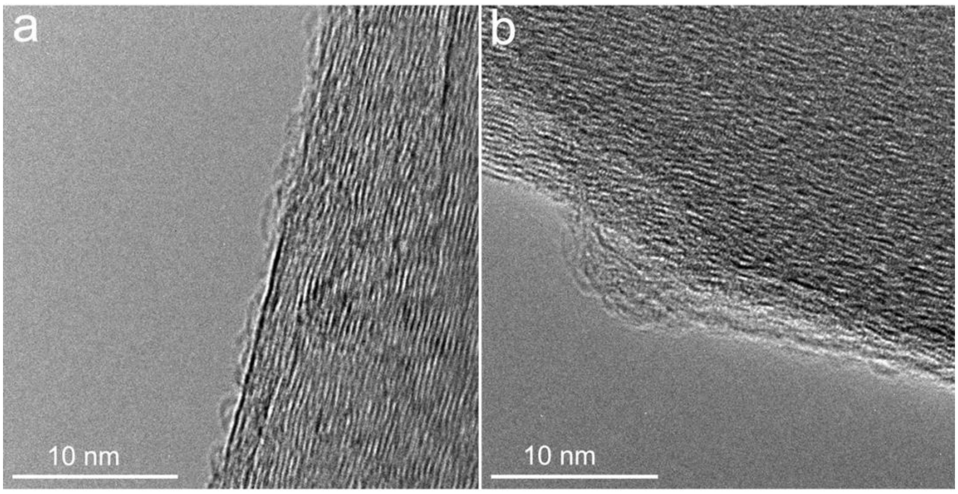

Figure S16. The HRTEM images of artificial organic SEI film on a graphite anode. (a) After 5 cycles. (b) After 50 cycles.

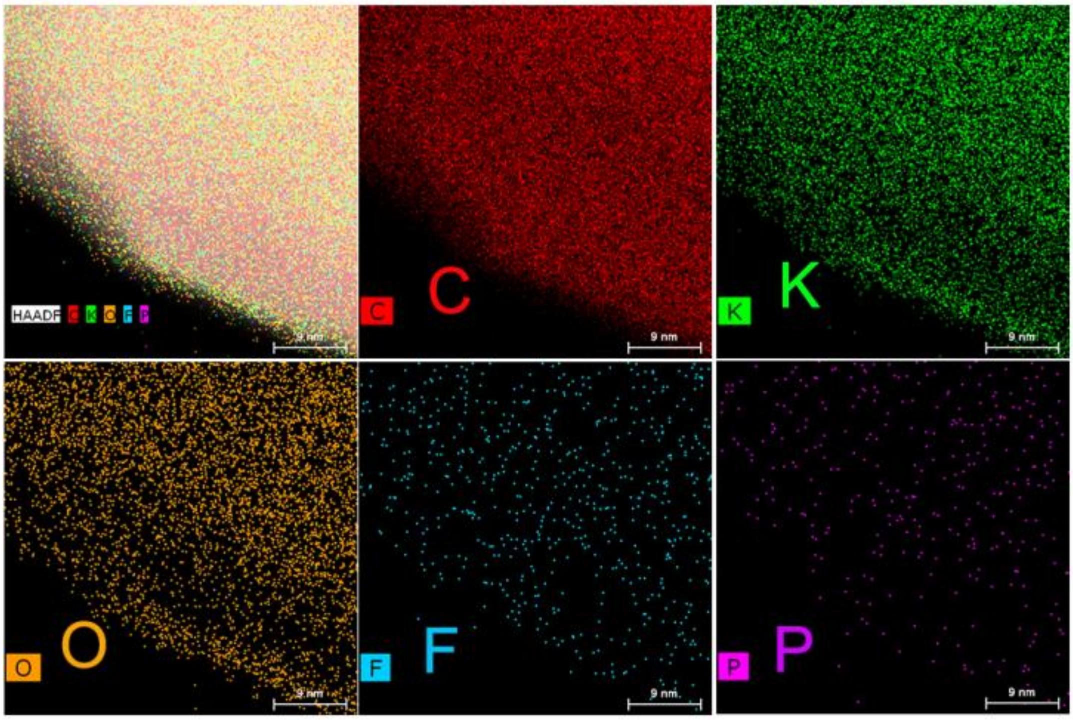

Figure S17. The elemental maps of the artificial organic SEI film prepared on a graphite anode surface by soaking it in KPF_6-electrolyte.

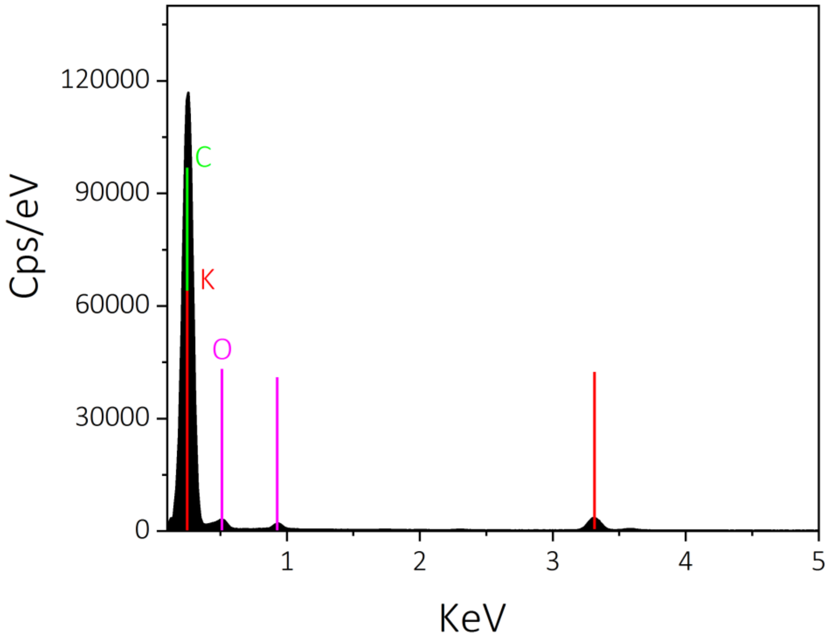

Figure S18. Energy spectrum of the traditional SEI film on a graphite anode.

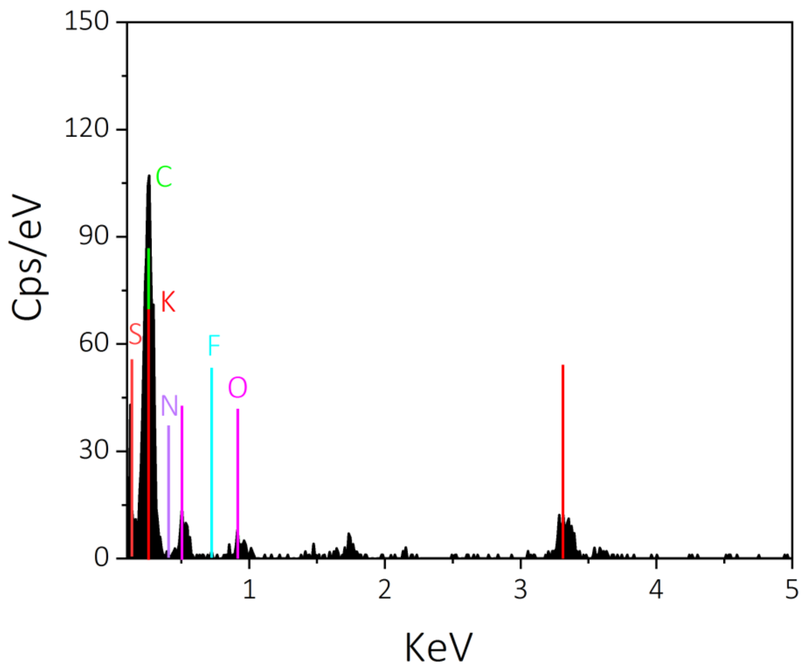

Figure S19. Energy spectrum of the artificial inorganic SEI film on a graphite anode.

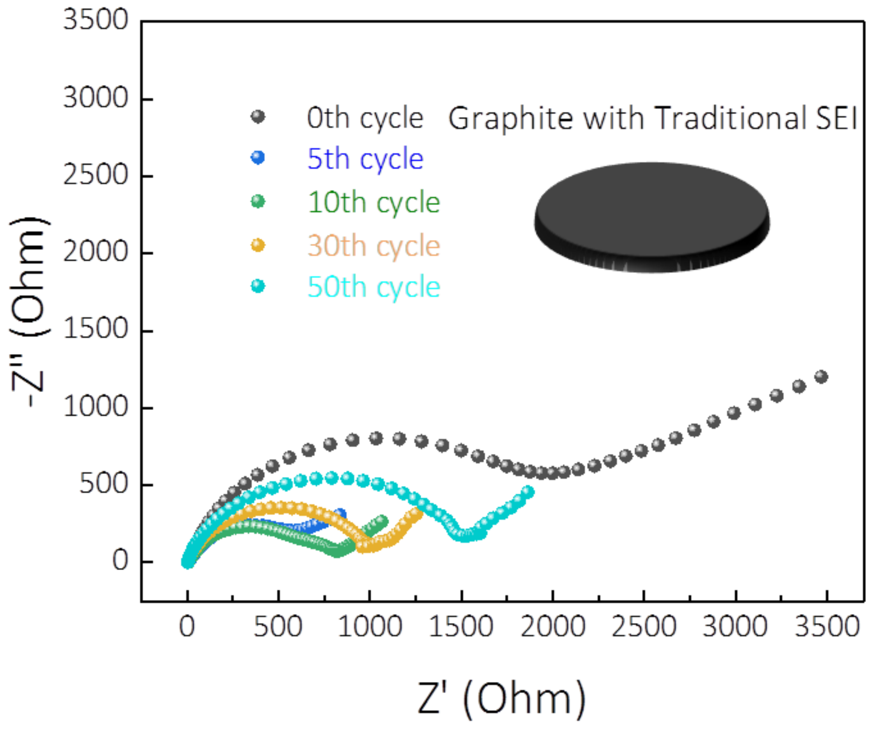

Figure S20. Nyquist plots of the traditional SEI film after 0, 5, 10, 30 and 50 cycles.

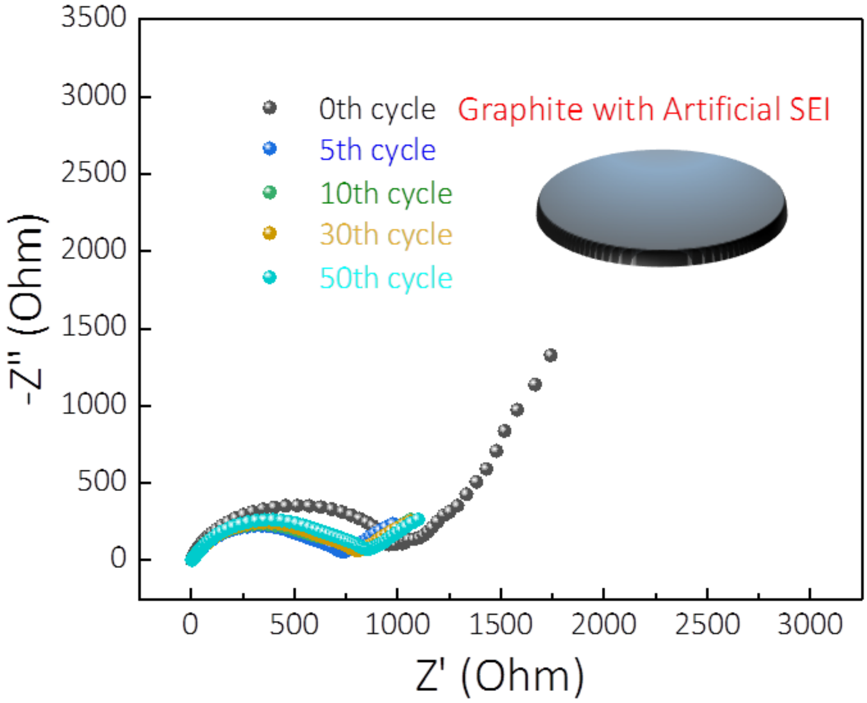

Figure S21. Nyquist plots of the artificial inorganic SEI film after 0, 5, 10, 30 and 50 cycles.

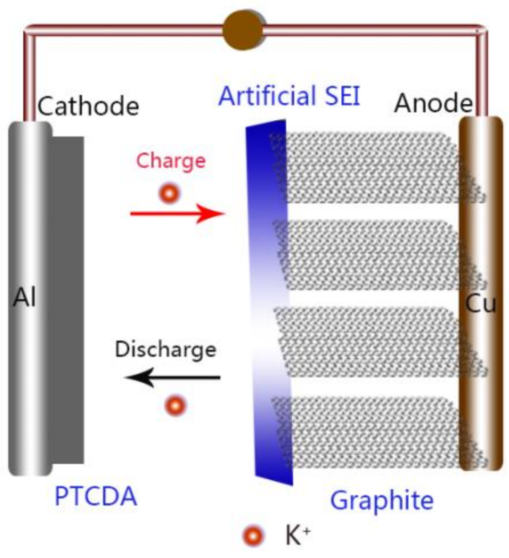

Figure S22. Schematic diagram of the full battery.

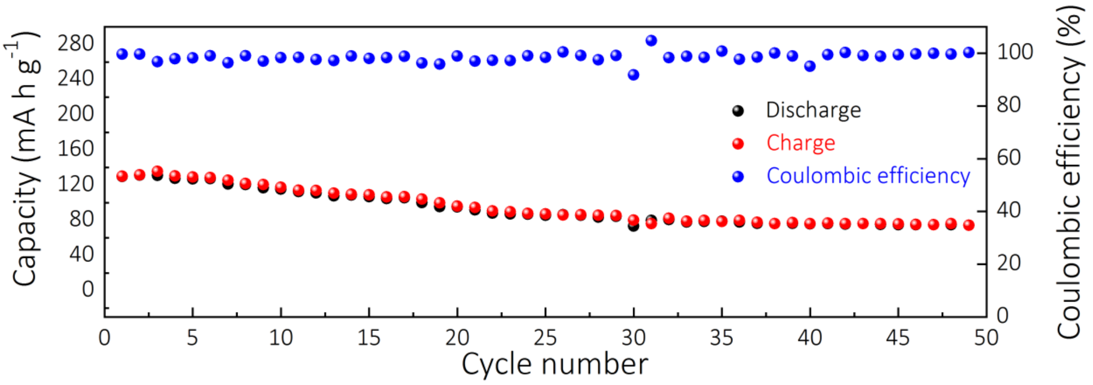

Figure S23. Cycle stability of PTCDA cathode in an organic electrolyte at 100 mA g^{-1}.
